# Physicians' explanatory behaviours and legal liability in decided medical malpractice litigation cases in Japan

**DOI:** 10.1186/1472-6939-12-7

**Published:** 2011-04-21

**Authors:** Tomoko Hamasaki, Akihito Hagihara

**Affiliations:** 1 Department of Nutirition Faculty of Home Economics, Kyushu Women's University 1-1 Jiyugaoka, Yahatanishi, Kitakyushu, Fukuoka, 807-8586, Japan; 2 Department of Health Services Management and Policy, Kyushu University Graduate School of Medicine, Higashi-ku, Fukuoka 812-8582, Japan

## Abstract

**Background:**

A physician's duty to provide an adequate explanation to the patient is derived from the doctrine of informed consent and the physician's duty of disclosure. However, findings are extremely limited with respect to physicians' specific explanatory behaviours and what might be regarded as a breach of the physicians' duty to explain in an actual medical setting. This study sought to identify physicians' explanatory behaviours that may be related to the physicians' legal liability.

**Methods:**

We analysed legal decisions of medical malpractice cases between 1990 and 2009 in which the pivotal issue was the physician's duty to explain (366 cases). To identify factors related to the breach of the physician's duty to explain, an analysis was undertaken based on acknowledged breaches with regard to the physician's duty to explain to the patient according to court decisions. Additionally, to identify predictors of physicians' behaviours in breach of the duty to explain, logistic regression analysis was performed.

**Results:**

When the physician's explanation was given before treatment or surgery (*p *= 0.006), when it was relevant or specific (*p *= 0.000), and when the patient's consent was obtained (*p *= 0.002), the explanation was less likely to be deemed inadequate or a breach of the physician's duty to explain. Patient factors related to physicians' legally problematic explanations were patient age and gender. One physician factor was related to legally problematic physician explanations, namely the number of physicians involved in the patient's treatment.

**Conclusion:**

These findings may be useful in improving physician-patient communication in the medical setting.

## Background

The physician's explanation to the patient and the patient's understanding of that explanation in the medical setting have become increasingly important in recent years. It has been reported that the physician's explanation and the level of the patient's understanding are related to patient satisfaction, patient treatment adherence, and treatment outcome [1-3]. Furthermore, it has been shown in recent years that inappropriate explanations by physicians can lead to medical disputes [4-9]. Thus, the physician's explanatory behaviour plays an important role in improving patient satisfaction, preventing medical disputes, and increasing treatment effectiveness.

The purposes of the physician's explanation are to obtain the patient's consent in cases of invasive care, to secure the patient's right of self-determination, to explain factors related to negative outcomes in patient care, and to give medical treatment guidance [10]. Legally, the physician's duty to explain is derived from the doctrine of informed consent and the physician's duty of disclosure. Thus, because the physician's explanation to the patient is legally required, an inappropriate or inadequate explanation is a breach of the duty of disclosure.

According to our survey on medical malpractice litigation in recent years in Japan, cases focusing on the physician's explanation to the patient have increased in number. When the physician delivers an insufficient explanation to the patient, the physician is deemed to have delivered substandard care, even if there is no fault with respect to his/her medical judgment or manual skills. This is because an insufficient explanation by a physician means a failure to fulfil the physician's duty derived from the medical contract between the physician and patient.

As a matter of course, relevant criteria for the level of a physician's explanation have become an issue. The first criterion is that the explanation should be as comprehensive as a typical doctor would provide in a similar situation (the "rational doctor criterion theory") [11-13]. The second criterion is that the doctor explains as much as the patient wants (the "concrete patient criterion theory") [11-13]. A third criterion consists of the first and second criteria in combination: the explanation is what a typical doctor would provide and as much as the patient wants to know.

Although these criteria may be useful in evaluating whether a physician's explanation to a patient is appropriate, they are still too abstract for practicing physicians. In fact, the abstract criteria concerning physicians' explanations to patients are likely not useful to health professionals engaged in medical practice. To date, no findings have addressed the specific physician explanatory behaviours by which possible breaches of the duty of disclosure can be evaluated in actual medical settings.

The number of medical malpractice claims has increased since the latter half of the 1990s in Japan, and this trend is partially due to breaches of the physician's duty of disclosure [14]. As noted, findings related to physicians' explanatory behaviours are extremely limited in Japan. Because decisions in litigated medical malpractice cases provide useful information about patient-physician interactions, we have analysed litigated medical malpractice cases in Japan [15-17]. We have examined the association between physicians' explanatory behaviours and physicians' legal liability, and have identified relationships between the specific manner of listening or talking to patients/families and decisions of negligent care [18]. However, several problems remain with respect to these findings. First, the decided cases analysed in those studies were not necessarily cases where the pivotal issue was the physician's duty of disclosure. Second, variables related to patient-physician interactions were extremely limited in number. Third, the number of decided medical litigation cases was not large enough to provide unbiased findings.

The present study takes the above issues into consideration and differs from our previous research as follows [15]. First, we have increased the number of decided medical malpractice cases in Japan where the main issue was the physician's duty to explain to the patient, by extending the time period in which decisions in medical litigation cases were made. Second, we have added more potential variables related to physicians' explanatory behaviours to make the selection of variables as comprehensive as possible. Examples of added variables include the number of times that the physician was present and whether consent was given by the patient or family.

Third, to identify predictors of physician behaviour regarding breach of the duty to explain, we have analysed of the patient and physician factors related to the physician's explanatory behaviour.

Using our dataset, we identified specific physician explanatory behaviours that may be related to the physicians' legal liability. Our findings may be useful for improving physicians' explanations to patients in medical settings.

## Methods

### (1) Data Source

We analysed legal decisions of medical malpractice cases. Specifically, decisions were collected for litigated medical malpractice cases reported between 1990 and 2009 in the *Hanrei Jiho *and *Hanrei Taimuzu*, major case records that report decided litigated cases in Japan; the cases examined were those in which the pivotal issue was the physician's duty to explain (366 cases).

Under the direction of one of the authors (TH), one graduate student and two students at Kyushu Dental College carefully read the decisions. Before reading the decisions, sessions were held to educate the students on the structure of a decision form, variables related to physician explanations, and patient and physician factors. One of the authors (TH) read all the decisions, and each student carefully read about one-third of all the decisions included in the analysis. After completing the reading of the decisions, the content of each decision was summarised using the study variables, and a database comprising the content of each decision (*n *= 366) was constructed. To verify the validity of data coding, kappa measures of agreement were calculated with respect to the nine variables related to the physician's explanation. With respect to the nine variables, shown in Table 1, kappa measures of inter-rater agreement between one of the authors (TH) and the three students were calculated. We obtained values of 0.83, 1.0, and 1.0 for the first variable (Purpose of explanation); 0.93, 0.97, and 0.88 for the second variable (Issue regarding the physician's explanation: If the physician's explanation was wrong, insufficient, or absent); 0.94, 0.94, and 0.85 for the third variable (Timing of the physician's explanation); 0.94, 1.00, and 0.81 for the fourth (Who received the physician's explanation); 0.83, 0.97, and 0.88 for the fifth variable (Manner of the physician's explanation to the patient: If the physician's explanation was delivered to a patient with or without a document, pamphlet, x-rays, model, or other explanatory material); 0.90, 1.0, and 0.88 for the sixth variable (Manner of the physician's explanation to the family: If the physician's explanation was delivered to the family with or without a document, pamphlet, x-rays, model, or other explanatory material); 0.78, 1.00, and 1.00 for the seventh variable (Level of the physician's explanation to the patient: If the physician's explanation to the patient was sufficiently relevant and specific); 0.88, 1.00, and 1.00 for the eighth variable (Level of the physician's explanation to family: If the physician's explanation to the family was sufficiently relevant and specific); and 0.87, 0.81, and 0.83 for the ninth variable (Location of the physician's explanation). These findings indicated good inter-rater agreement. In cases in which coding differed between raters, the cases were discussed based on the coding criteria, and a consensus was reached.

**Table 1:** Profile of the study variables concerning physician's explanatory behaviours (*n *= 366)

Items	Number of cases (%)	
Purpose of the explanation	Explanation to obtain the patient's consent	213 (67.0)
	Other ^a^	105 (33.0)
Issue of the physician's explanation	No explanation	61 (19.2)
	Incorrect or insufficient explanation	257 (80.8)
Timing of the physician 's explanation	Before treatment or surgery	203 (79.0)
	After treatment or surgery	54 (21.0)
Who received the physician's explanation	Patient only	99 (38.4)
	Patient and family	93 (36.0)
	Family only	66 (25.6)
Manner of the physician 's explanation to the patient	Oral only	144 (75.8)
	Oral and other methods	46 (24.2)
Manner of the physician 's explanation to family	Oral only	129 (81.6)
	Oral and other methods	29 (18.4)
Level of the physician's explanation to the patient	Relevant and specific	60 (33.0)
	Not sufficiently relevant or specific	122 (67.0)
Level of physician's explanation to family	Relevant and specific	49 (36.3)
	Not sufficiently relevant or specific	86 (63.7)
Location of physician's explanation	Inpatient ward	173 (54.4)
	Outpatient clinic	145 (45.6)
Content of the physician's explanation	Related to surgery	132 (36.1)
	Other ^b^	234 (63.9)
Number of times that the physician explained	Once	109 (43.1)
	Twice or more	144 (56.9)
Consent by the patient	With the patient's consent	151 (90.4)
	Without the patient's consent	16 (9.6)
Consent by family	With family consent	113 (91.9)
	Without family consent	10 (8.1)
Written consent by the patient	Presence	45 (52.3)
	Absence	41 (47.7)
Written consent by family	Presence	37 (53.6)
	Absence	32 (46.4)
Day of the physician's explanation	Same day as treatment	41 (21.1)
	Not the same day	153 (78.9)

### (2) Study Variables

Table 2 shows the variables related to patient and physician characteristics. Of the patient characteristics, "type of treatment" had two subcategories: "elective or not urgently necessary" and "others".

**Table 2:** Profile of the study variables related to patients and physicians (*n *= 366)

	Items	Number of cases (%) or mean ± SD (*n*)	
Patient characteristics	Age (years)	37.15 ± 24.44(316)	
	Gender	Male	185 (51.5)
		Female	174 (48.5)
	Type of treatment	Elective or not urgently necessary	33 (9.0)
		Other ^a^	330 (91.0)
	Severity of injury	Death	160 (43.7)
		Other ^b^	206 (56.3)
	Patient's fault or treatment refusal	Presence	18 (4.9)
		Absence	145 (95.1)
	Question from the patient	Yes	100 (28.5)
		No	251 (71.5)

Physician characteristics	Department where patients were treated	Surgical system	34 (34.0)
		Other ^c^	66 (66.0)
	Type of medical facility	Clinic	79 (21.7)
		Hospital	285 (78.3)
	Number of physicians	1	195 (53.3)
		2 or more	170 (46.4)
	Gender	Male	294 (90.7)
		Female	30 (9.3)
	Medical standard	Standard care	302 (83.2)
		Not standard care	61 (16.8)

a: "Other" includes "treatment is urgently necessary"(n = 6) and "other" (n = 324). b: "Other" includes temporary or permanent injury. c: "Other" includes internal medicine, paediatrics, obstetrics, ophthalmology, otolaryngology, dermatology, dentistry, urology, and other departments.

The physician's duty to provide an explanation to the patient is severely judged in the field of cosmetic surgery, where treatment is elective [19]. There was a difference between cosmetic surgery and other medical treatments in terms of the criteria for the physicians' explanation to patients. Thus, these two categories were created for "type of treatment". "Severity of injury" was subdivided into the categories of "death" and "others". "Others" includes temporary or cured injury and permanent or uncured injury.

It has been reported that poor patient-physician communication was predictive of medical claims among internists, but not among surgeons [7]. Based on this finding, "department where patients were treated" was split into two subcategories, "surgical system" and "others". The type of medical facility was classified as "clinic" or "hospital" based on the findings in the decision specifying that, according to medical law in Japan, "a medical institution having hospitalisation facilities with more than 20 beds" is defined as a hospital, and "a medical institution having hospitalisation facilities with 19 beds or fewer" is defined as a clinic (Medical Law, Article 5, Law No. 205, 1948). When a treatment in question has not been established as a medical standard at the time of the treatment, the level of the physician's explanation about the treatment is not required to be as high as in cases for which standard care has been defined. "Medical standard" was a court judgment with respect to whether a treatment was established as a medical standard, and two judgements were possible: "standard care" and "not standard care".

Table 3 lists variables related to the physician's explanatory behaviour. "Purpose of explanation" has two categories: "explanation to obtain patient's consent" and "others". As a general rule, the purposes of physician explanations are [1] to obtain the patient's consent, [2] to provide guidance for medical treatment, and [3] to provide post-treatment explanations. Of these, the explanation given to obtain patient's consent is related to the patient's right of self-determination, indicating that explanation for this purpose differs from other purposes. Thus, "purpose of explanation" was split into the two subcategories of "explanation to obtain the patient's consent" and "others". "Timing of physician's explanation" was divided into two categories according to when the explanation was given: "before treatment or surgery" and "after treatment or surgery".

**Table 3:** Number of cases according to acknowledgement of physician liability in the court decision.

Court decision	Judgment reason	N (cases)
Acknowledged physician liability		
	Acknowledgement of a breach of physician's duty to explain	145
	Acknowledgement of the physician's fault only	71
	
	Subtotal	216

No acknowledged physician liability		150

Total		366

A, "Other" includes explanation about medical treatment guidance and explanation about reasons for negative outcomes. B, "Other" includes explanations about medical treatment and medical testing.

In Japanese medical settings, the family tends to play an important role when receiving the physician's explanation. Thus, "who received the physician's explanation" had three categories: "patient only", "patient and family", and "family only". "Manner of physician's explanation to patient" and "manner of physician's explanation to family" were both subdivided into two categories: "oral only" and "oral and other methods". Other methods included documents and pamphlets. With regard to the "level of the physician's explanation to the patient" and "level of the physician's explanation to family", the explanations were classified as "relevant and specific" or "not sufficiently relevant or specific" according to the raters' judgments about the relevancy and specificity of the explanation.

"Location of physician's explanation" was classified as "inpatient ward" or "outpatient clinic". In cases where treatment is closely related to the patient's life or health, it is generally recognised that a doctor needs to explain fully what is happening to the patient. Therefore, "content of the physician's explanation" was categorised into "related to surgery" or "others". The matter of consent by the patient or family had two categories, "with consent" and "without consent", applied to the cases of patient and family consent. With respect to written consent by the patient or family, when the presence of a consent *document *was clear, consent was classified as "presence". Finally, "the day of physician's explanation" refers to the day when the physician's explanation was completed, and this was categorised as "the same day" if the physician's explanation was completed on the same day that the surgery or treatment was performed and "not the same day" if the physician's explanation was completed before the day that the surgery or treatment was performed.

### Statistical Analyses

To evaluate associations among the patient characteristics, physician characteristics, physician's explanatory behaviour, and physician's duty to explain, Student's *t*-test for continuous variables and the χ^2 ^test for categorical variables were used. To identify factors related to the breach of a physician's duty to explain, an analysis was undertaken by acknowledged breach status with regard to the physician's duty to explain to the patient according to the court decisions. Additionally, to identify predictors of physicians' behaviour in breach of the duty to explain, logistic regression analysis was performed. In the analysis, cases in which the patients were 14 years of age or younger were excluded. This was because, in these cases, the recipient of the physician's explanation was likely the patient's parents. Although patient's age is shown to be a significant predictor of a physician's explanatory behaviour, it would be difficult to interpret the result when including such young patients. The statistical software package PASW Statistics (for Mac, ver. 18) was used for the analysis.

## Results

Table 3 shows the number of cases by the type of court decision. Physician's liability was found by the court in 216 cases, and no breach was found in 150. In the former group, "acknowledgement of breach of physician's duty to explain" was found by the court in 145 cases, and "acknowledgement of the physician's fault only" was found in 71 cases. "Physician's fault" is defined as a physician's mistake in technical performance, an error in the physician's judgment, or both.

Table 2 shows the prevalence of all study variables. The mean age of the patients was 37.2 years, and 51.5% of them were males. Elective or not urgent or necessary treatments constituted 9% of all cases, and necessary treatments made up 91%. With regard to the severity of injury, 43.7% of all cases resulted in death. The proportion of all cases that involved patient questions was 28.5%. The surgical department was involved with patient care in 34% of all cases. Concerning the type of medical facility, the proportions of hospitals and clinics were 78.3% and 21.7%, respectively. The proportion of patient care involving two or more physicians was 46.4% of all cases.

Table 1 lists variables related to physicians' explanatory behaviour. Concerning the purpose of physicians' explanations, 67.0% constituted explanations to obtain the patient's consent. While the majority of physician explanations made to obtain patient consent were delivered to the patients before treatment, a few explanations were made after giving emergency treatment. With respect to the timing of the explanation, 79.0% of explanations were given before treatment or surgery. The physician's explanation was given to the patient alone in 38.4% of all cases, and to both the patient and family or to family alone in 61.6% of all cases. With regard to the manner of the explanation, an oral explanation alone was provided in 75.8% of all cases, whereas explanations used oral and other methods in 24.2% of all cases. With respect to the physician's explanation to the family, oral explanation alone accounted for 81.6% of cases. Concerning the level of the physician's explanation to the patient, 33.0% of cases involved relevant and specific information. The explanation occurred in the inpatient ward in 54.4% of cases, and the content of the physician's explanation was related to surgery in 36.1% of cases. The proportion of times when the physician explained just once was 43.1%. The patient's consent was obtained in 90.4% of all cases, and written consent was obtained in 52.3% of all cases. Finally, the physician's explanation occurred on the same day as treatment/surgery in 21.1% of all cases.

Table 4 shows the proportion of all explanations represented by each of the 16 explanatory behaviours, indicated in parentheses, among negligent and non-negligent groups. With regard to the timing of the physician's explanation, the proportion of explanations provided before treatment or surgery was significantly greater in the "yes" than in the "no" group (*p *= 0.006). With regard to the level of the physician's explanation to the patient and to the family, the proportion of "relevant and specific" explanation was significantly greater in the "yes" group than in the "no" group (*p *< 0.001 for both). With respect to the location of the physician's explanation, the proportion of "outpatient ward" was significantly greater in the "yes" group than in the "no" group (*p *< 0.001). Regarding patient's consent, the proportion of treatments carried out without a patient's consent was significantly greater in the "yes" group than in the "no" group (*p *= 0.002). With regard to the timing of the physician's explanation, the proportion of explanations occurring on the same day was significantly larger in the "yes" group than in the "no" group (*p *= 0.004).

**Table 4:** Comparison of the physician's explanatory behaviours between negligent and non-negligent groups concerning the physician's duty to explain: n (%)

Physician's explanatory behaviours	Court decision with respect to a physician's duty to explain		***p*-value**^**a**^
		
	Negligent	Non-negligent	
Purpose of the physician's explanation (explanation to obtain patient's consent)	91 (62.8%)	121 (70.3%)	0.153
Issue of the physician's explanation (no explanation)	26 (17.9%)	35 (20.3%)	0.586
**Timing of the physician's explanation (before treatment or surgery) **	**85 (71.4%)**	**117 (85.4%)**	**0.006**
Who received the physician's explanation (patient only)	50 (42.0%)	49 (35.5%)	0.285
Manner of the physician's explanation to the patient (oral only)	70 (77.8%)	73 (73.7%)	0.518
Manner of the physician's explanation to family (oral only)	60 (85.7%)	68 (78.2%)	0.225
**Level of the physician's explanation to the patient (relevant and specific) **	**0 (0.0%)**	**59 (65.6%)**	**0.000**
**Level of the physician's explanation to family (relevant and specific) **	**1 (1.5%)**	**47 (69.1%)**	**0.000**
**Location of the physician's explanation (inpatient ward)**	**62 (42.8%)**	**110 (64.0%)**	**0.000**
Content of the physician's explanation (related to surgery)	46 (31.5%)	63 (36.4%)	0.357
Number of times that the physician explained (once)	49 (41.9%)	59 (44.4%)	0.693
**Consent by the patient (with the patient's consent) **	**61 (82.4%)**	**88 (96.7%)**	**0.002**
Consent by family (with family's consent)	45 (93.8%)	66 (90.4%)	0.384
Written consent by the patient (presence)	22 (45.8%)	22 (59.5%)	0.213
Written consent by family (presence)	15 (41.7%)	21 (56.8%)	0.491
The day of physician's explanation (not the same day)	57 (69.5%)	93 (85.3%)	0.008

^a ^*t*-test or χ^2 ^test; results printed in bold are significant (*p *< 0.05)

Finally, because six explanatory physician behaviours related to the legal responsibility to explain to the patient were assessed, to identify relevant patient or physician characteristics, we performed logistic regression analyses with patient and physician characteristics as explanatory variables and the physician's explanatory variables as dependent variables (Table 5). As explained in the Method section, cases in which the patients were 14 years of age or younger (23% of all cases) were excluded from the analysis.

**Table 5:** Stepwise logistic regression analyses of factors related to the physician's explanatory behaviour

Purpose variable	Explanatory variables	β	*p-*value	95%CI
Timing of physician 's explanation (0: before treatment or surgery, 1: after treatment or surgery	Patient age	1.002	0.891	0.974-1.030
	**Patient gender **(0: male, 1: female)	**0.391**	**0.039**	**0.160-0.954**
	Type of treatment (0: elective or not urgently necessary, 1: others)	0.336	0.192	0.065-1.726
	Question from the patient (0: yes, 1: no)	0.625	0.303	0.255-1.531
	**Number of physicians **(0: 1, 1: 1 or more)	**0.221**	**0.001**	**0.088-0.555**
	Medical standard (0: standard care, 1: not standard care)	0.226	0.171	0.027-1.898

Level of the physician's explanation to the patient (0: relevant and specific, 1: not sufficiently relevant or specific)	**Patient age**	**0.962**	**0.004**	**0.936-0.988**
	Patient gender (0: male, 1: female)	0.835	0.650	0.384-1.817
	Type of treatment (0: elective or not urgently necessary, 1: others)	0.948	0.937	0.248-3.624
	Question from the patient (0: yes, 1:no)	0.588	0.225	0.249-1.387
	**Number of physicians **(0: 1, 1: 1 or more)	**0.355**	**0.012**	**0.159-0.793**
	Medical standard (0: standard care, 1: not standard care)	4.194	0.076	0.833-20.51

Level of the physician's explanation to family (0: relevant and specific, 1: not sufficiently relevant or specific)	**Patient age**	**0.968**	**0.022**	**0.941-0.995**
	Patient gender (0: male, 1: female)	0.632	0.332	0.250-1.597
	Type of treatment (0: elective or not urgently necessary, 1: others)	1.174	0.904	0.085-16.21
	Question from the patient (0: yes, 1:no)	0.653	0.403	0.241-1.773
	**Number of physicians **(0: 1, 1: 1 or more)	**0.319**	**0.035**	**0.110-0.925**
	Medical standard (0: standard care, 1: not standard care)	1.196	0.779	0.432-13.83

Location of physician's explanation (0: inpatient ward, 1: outpatient clinic)	Patient age	0.996	0.708	0.976-1.016
	Patient gender (0: male, 1: female)	1.173	0.632	0.612-2.249
	Question from the patient (0: yes, 1: no)	1.941	0.079	0.927-4.064
	**Number of physicians **(0: 1, 1: 1 or more)	**0.231**	**0.000**	**0.120-0.443**
	Medical standard (0: standard care, 1: not standard care)	0.517	0.220	0.180-1.483

Consent by the patient (0: with the patient's consent, 1: without the patient's consent	Patient age	0.971	0.147	0.934-1.010
	**Patient gender (0: male, 1: female)**	**6.223**	**0.022**	**1.305-29.67**
	Type of treatment (0: elective or not urgently necessary, 1: others)	0.187	0.139	0.020-1.726
	Question from the patient (yes/no)	0.858	0.804	0.255-2.883
	Number of physicians (0: 1, 1: 1 or more)	1.118	0.854	0.340-3.672
	Medical standard (0: standard care, 1: not standard care)	0.852	0.850	0.163-4.456

The day of the physician's explanation (0: not the same day, 1: the same day)	**Patient age**	0.974	0.089	0.945-1.004
	Patient gender (0: male, 1: female)	1.878	0.182	0.745-4.733
	Type of treatment (0: elective or not urgently necessary, 1: others)	0.732	0.616	0.216-2.477
	Question from the patient (0: yes, 1:no)	2.239	0.115	0.821-6.107
	**Number of physicians **(0: 1, 1: 1 or more)	**0.255**	**0.004**	**0.101-0.643**
	Medical standard (0: standard care, 1: not standard care)	0.742	0.682	0.178-3.091

Results printed in bold are significant (*p *< 0.05).

Consequently, "number of the physicians" was found to be significantly associated with the physician's explanatory behaviours. Additionally, "patient age" and "patient gender" were also significantly associated with the physician's explanatory behaviours (Table 5).

## Discussion

In this study of decided litigated medical malpractice cases where the main issue was the physician's duty to explain in Japan, we examined the association between the physician's explanatory behaviour and the legal responsibility to explain to the patient. In addition, after identifying physicians' explanatory behaviours that were deemed to be a breach of the duty to explain, we identified patient and physician characteristics related to such explanatory behaviours.

Before discussing our findings, we should examine the validity of data coding under the direction of one of the authors (TH). Although dental students read and coded the decisions, we believed that there would be no problems with respect to the coding procedure for the following reasons. First, one of the authors (AH), whose specialty is medical law, conducted sessions to educate the students on the structure of a decision form and variables related to physician explanations before reading the decisions. Second, the variables in the decisions were checked and coded using objective criteria. Third, kappa measures of agreement between one dental student and one of the authors (TH) had very high values, indicating that the coding was valid.

### *1*. Association between physicians' explanatory behaviour to patients and legal liability

Several notable findings were obtained in the study. First, the proportion of cases that included patient consent was significantly larger in the group in which no breach of the physician's duty to explain was found compared to the group in which a breach of that duty was found (Table 4). Interestingly, written consent by the patient or family showed no relationship to the physician's legal liability (Table 4). Courts in the US have also decided that the patient's or family's written consent is not necessarily evidence in support of the physician in medical malpractice litigation. A pivotal issue in deciding whether a defendant is negligent is whether the physician provided an explanation to the patient in response to the patient's questions in the case [20]. The present findings are consistent with the US situation. The result suggests that the patient's actual consent is more important than a particular written consent form.

Second, the frequency of physicians' explanations was related to the legal responsibility to explain. However, when the physician's explanation and the treatment/surgery were on the same day, the explanatory behaviours were more likely to be deemed a breach of the duty to explain (Table 4). This suggests that it is important to give patients sufficient time to consider treatment options after they receive explanations from the physician. In these situations, the physician may be rushed and too busy to explain the treatment options well. Since, in many cases, the patient has little medical knowledge and may be mentally or physically unstable, it is difficult for the patient to decide on a treatment option adequately in a short period time. Therefore, where possible, the physician should avoid performing medical treatment or surgery on the day of providing explanations to the patient. In reality, however, same-day treatment is often necessary when dealing with emergency cases. Since doctors have to give explanations to patients after treatment in emergency cases, they need to recognise the risks accompanying post-treatment explanation and try to provide an explanation that is specific and relevant to the treatment and disease.

Next, our results showed that the proportion of acknowledged breaches of the physician's duty to explain was low when an explanation was given before treatment or surgery (Table 4). This shows the importance of explaining a procedure or regimen before performing or administering it. Moreover, although irrelevant or non-specific physician explanations to the patient or family were more likely to be judged a breach of the physician's duty to explain, the manner of the physician's explanation (e.g., oral, written) showed no relationship with court decisions (Table 4).

With respect to the *content *of physician explanations, various views have been offered. For example, in the UK, where physicians are only required to give general or non-specific explanations, the demand for relevant or specific explanations has been increasing in recent years [21]. In Japan, the level of explanation by physicians has been left to the physician's discretion. However, in a recent case of medical malpractice litigation, it was decided that the physician's explanation needs to be specific or sufficiently relevant to the patient's concerns, such as concerns regarding the treatment and disease [22]. This decision seems to have affected recent medical malpractice litigation in Japan. Our findings show that the physician's explanation to the patient should be specific or relevant (Table 4), consistent with the cited decision.

### *2) *Patient and physician characteristics related to legally problematic explanations

Few studies have examined the association between patient attributes and legally problematic explanatory behaviours by physicians in medical litigation [23]. In the present study, patient age and gender were associated with legally problematic explanatory behaviours. First, with regard to age, the older the patient was, the more common it was that the physician's explanation was specific (Table 5). This suggests that physicians are more likely to deliver incomplete explanations, consciously or unconsciously, when patients are younger. As a practical implication, physicians should try to provide the same explanation regardless of the age of the patient, and young patients should be urged to ask for further details when they do not understand the physician's explanation. Another explanation is also possible, namely that elderly patients might not complain more than younger patients in a medical setting, thus prompting more explanation from their physicians. In this case too, a practical implication is that physicians should try to provide the same explanation regardless of the patient's age.

Second, patient gender was a significant predictor of patient consent (Table 5). Treatment or surgery was performed without the patient's consent more frequently in female patients. This might be because many female patients were treated during labour and delivery. In many of these cases, it was decided that physicians should have obtained consent from the patient, *not *from the family, immediately after the birth. It has been reported that communication is an important factor in decreasing the amount of litigation in obstetrics and gynaecology [24]. Although the number of medical disputes is consistently high in obstetrics and gynaecology, it may be possible to reduce litigation by obtaining the patient's consent immediately after childbirth, at least when the pivotal issue in a case is the physician's duty to explain.

Next, we consider the physician characteristics. First, breach of duty was more likely to be found when two or more physicians were involved in treating the patient, and less likely when the medical facility was a hospital, when the timing of the physician's explanation was before treatment or surgery, when the physician's explanation to the patient and family was relevant or specific, when the location of the physician's explanation was the inpatient ward, and when the day of the physician's explanation was not the same as the day of treatment or surgery (Table 5). These findings might indicate that there is a difference between hospitals and clinics in terms of the content of medical care and the demographic characteristics of patients. The importance of the physician's explanation appears to be better recognised and is more strictly performed in hospitals than in clinics.

## Limitations of the study and future problems

Finally, we discuss the limitations of this study and future problems. First, this study does not deal with *all *recent court decisions concerning violations of the physician's duty to explain during the study period in Japan. Therefore, bias may have been introduced because the decisions were published in case reports according to topicality and a new interpretation of the laws. In fact, decisions that favoured patients in general medical malpractice litigation in Japan between 1976 and 1987 represented only 37.3% of cases [16], whereas our study showed 59%. This difference in the proportion of decisions in favour of patients suggests the importance of the physician's explanation to the patient. That is, cases involving violations of the physician's duty to explain are more likely to be decided in the patient's favour. However, because the type of decisions used for the analysis here may differ from the substance of decided medical disputes in general, a simple comparison of the proportions might not be meaningful. Thus, caution is needed with regard to the external validity of these findings.

Despite these problems, factors from medical disputes revealed on the basis of litigated medical malpractice cases have practical implications. Few findings have been based on quantitative analyses of decided medical malpractice litigations in Japan. In addition, because decided cases in which the pivotal issue was the physician's duty to explain were analysed in this study, our findings have practical implications because the physician's duty to explain to the patient may become an important issue in patient-physician interactions. Future studies are needed to confirm the validity of our results.

## Conclusions

The following points were demonstrated in our study. (1) When the physician's explanation was given before treatment or surgery, when the physician's explanation was relevant or specific, and when the patient's consent was obtained, the explanation was less likely to be deemed a breach of the physician's duty to explain. (2) The manner of the physician's explanation, frequency of the physician's explanations, and written consent by the patient or family showed no relationship to the court decisions. (3) Patient factors related to legally problematic explanations were patient age and gender. (4) A physician factor related to legally problematic explanations was the number of physicians involved in treating the patient. In conclusion, characteristic physician explanatory behaviours related to the legal duty to explain to the patient were revealed. Physicians should be aware of factors associated with the breach of duty to explain, including patient's gender and age, the number of treating physicians, the size of the medical facility, and whether an explanation is given before or on the day of treatment. These findings may help physicians provide appropriate explanations to their patients.

## Competing interests

The authors declare that they have no competing interests.

## Authors' contributions

TH gathered data, conducted the statistical analysis, and drafted the manuscript. AH was responsible for the scientific direction and design of the study and assisted with editing the paper. All authors read and approved the final manuscript.

## Pre-publication history

The pre-publication history for this paper can be accessed here:

http://www.biomedcentral.com/1472-6939/12/7/prepub
